# Sharing clinical trial data on patient level: Opportunities and challenges

**DOI:** 10.1002/bimj.201300283

**Published:** 2014-06-18

**Authors:** Franz Koenig, Jim Slattery, Trish Groves, Thomas Lang, Yoav Benjamini, Simon Day, Peter Bauer, Martin Posch

**Affiliations:** 1 Center for Medical Statistics, Informatics and Intelligent Systems, Medical University of ViennaSpitalgasse 23, 1090, Vienna, Austria; 2 European Medicines Agency (EMA)7 Westferry Circus – Canary Wharf, London, E14 4HB, UK; 3 BMJLondon, WC1H 9JR, UK; 4 AGES – Austrian Agency for Health and Food Safety, Federal Office for Safety in Health CareTraisengasse 5, 1200, Vienna, Austria; 5 Department of Statistics and Operations Research, The Sackler Faculty of Exact Sciences, Tel Aviv UniversityTel Aviv, 6997801, Israel; 6 Clinical Trials Consulting and Training53 Portway, North Marston, Buckinghamshire, MK18 3PL, UK

**Keywords:** EMA draft policy/0070, Open access to clinical trial data, Raw data, Secondary research, Transparency, Validation

## Abstract

In recent months one of the most controversially discussed topics among regulatory agencies, the pharmaceutical industry, journal editors, and academia has been the sharing of patient-level clinical trial data. Several projects have been started such as the European Medicines Agency´s (EMA) “proactive publication of clinical trial data”, the *BMJ* open data campaign, or the AllTrials initiative. The executive director of the EMA, Dr. Guido Rasi, has recently announced that clinical trial data on patient level will be published from 2014 onwards (although it has since been delayed). The EMA draft policy on proactive access to clinical trial data was published at the end of June 2013 and open for public consultation until the end of September 2013. These initiatives will change the landscape of drug development and publication of medical research. They provide unprecedented opportunities for research and research synthesis, but pose new challenges for regulatory authorities, sponsors, scientific journals, and the public. Besides these general aspects, data sharing also entails intricate biostatistical questions such as problems of multiplicity. An important issue in this respect is the interpretation of multiple statistical analyses, both prospective and retrospective. Expertise in biostatistics is needed to assess the interpretation of such multiple analyses, for example, in the context of regulatory decision-making by optimizing procedural guidance and sophisticated analysis methods.

## 1 Introduction

In November 2012 Dr. Guido Rasi, the current executive director of the European Medicines Agency (EMA), announced that “*…we are not here to decide if we publish clinical-trial data, but how*!” at an EMA workshop on clinical trial data and transparency. Furthermore, he stated that “*we need to do this in order to rebuild trust and confidence in the whole system*”. This meeting can be considered as a milestone in EMA's activities^1^ on transparency and release of clinical trial data. In principle any EU citizen can request any document s/he is interested in from any EU institution (see Article 255 of the treaty establishing the European Community), including from the EMA. However, in the past the EMA was heavily criticized on several occasions for not releasing (sufficient) information after external requests. Also, under pressure from the European Ombudsman, who considered the EMA's repeated refusals to disclose public documents as constituting acts of “maladministration”, the EMA published a new access-to-documents policy in 2010 (EMA, 2010). This change in its transparency policy may be considered as a significant step toward greater transparency, as the EMA has released more than 1.9 million pages in response to such requests since November 2010.

Just before the MCP meeting in July 2013 (Wei et al., 2014) the EMA released its draft policy/0070 on “publication and access to clinical trial data” for public consultation (EMA, 2013). The initial plan of the EMA was to publish the final policy at the end of 2013, coming into force on January 1, 2014. As the draft policy promoted such a huge debate among all stakeholders, the initial timelines were not met. In a press release, the EMA reinforced its commitment “to push ahead in 2014 toward publication and access to clinical trial data”, but announced a stepwise approach and implementation to be developed in the first half of 2014 (EMA Press Office, 2013). The EMA draft policy acknowledges the importance of personal data protection when releasing clinical trial data but the mechanism by which an acceptable level of protection can be achieved is, as yet, unclear. Open access means that such data will be available as downloads from the EMA webpage. This includes the clinical study report— meaning all elements submitted as study reports in CTD Module 5, following the format of the ICH E3 document—redacted to exclude identifiable personal information. Furthermore, in the draft policy the EMA suggests “controlled access” to raw clinical trial data. This includes individual patient datasets, individual patient line-listings, individual case report forms, and documentation explaining the structure and content of datasets. Controlled access means that a specific request has to be sent to the EMA and that data would only be released after a decision regarding a marketing authorization had been reached.

Besides the EMA, other initiatives on transparency have been launched in recent years. The US FDA have discussed the availability of de-identified and masked data derived from medical product applications (FDA, 2013). One of the most prominent initiatives is that of AllTrials, which demands that “all trials past and present should be registered, and the full methods and the results reported” (see http://www.alltrials.net/). Even today, a third of clinical trials have not published results five years after they ended (Jones et al., 2013). Also medical journals insist on more transparency before publishing papers, for example, according to the *BMJ* Open Data Campaign the *BMJ* will no longer publish any trial of drugs or devices where the authors do not commit to making the relevant anonymized patient-level data available, upon reasonable request.

There are also individual proposals by pharmaceutical companies on how to share clinical trial data at the patient level, for example, the GlaxoSmithKline Data Transparency Initiative or Roche Global Policy on Sharing of Clinical Trial Data. In these proposals researchers may receive access to patient-level data after requests have been reviewed by an (independent) panel of experts. A joint statement was issued by EFPIA and PhRMA on “Principles for Responsible Clinical Trial Data Sharing” (EFPIA and PhRMA, 2013).

The EMA have stated that information that is currently deemed to be commercially confidential will be accessible via the EMA in the future (Eichler et al., 2012). However, two pharmaceutical companies (AbbVie and InterMune) have challenged the EMA's way to grant access to information (including clinical study reports) in accordance with its 2010 access-to-documents policy. In April 2013, the EMA was ordered by the General Court of the European Union not to provide documents until a final ruling is given by the Court. It is not clear how this interim court decisions might impact EMA's plan to provide access to data on patient level by next year.

All these initiatives will change the landscape of clinical development and publication of medical research. They provide unprecedented opportunities for research and research synthesis, but pose new challenges for regulatory authorities, sponsors, scientific journals and the public. An important issue in this respect is the interpretation of multiple statistical analyses (both, prospectively and retrospectively planned). How can the reliability of findings from such multiple analyses be assessed and optimized, for example, by procedural and analysis guidance?

In Section 2013, Franz Koenig, Peter Bauer, and Martin Posch elaborate on the two different main types of scientific research based on open access to patient-level data: validation of clinical trial results and reuse of data to investigate new scientific questions. In Section 3, Jim Slattery raises the question whether public access to data from randomized clinical trials will improve our understanding of drug safety. In Section 4, Trish Groves elaborates on what journals can do with regards to data sharing. In Section 5, Thomas Lang discusses the transparency initiative from the viewpoint of a national regulatory agency embedded in the EU regulatory system. In Section 6, Yoav Benjamini shows how the false discovery rate (FDR) can be used to tackle some of the multiplicity issues arising when reanalyzing data. In Section 7, Simon Day illustrates how the benefits of data sharing outweigh its risks from his perspective as former regulator, industry statistician, and current journal editor. Finally, in Section 8 we will give some concluding remarks.

## 2 Perspectives from Franz Koenig, Peter Bauer, and Martin Posch

Broadly speaking, the benefit of publication of clinical trial data at the patient level can be classified in two types of scientific research: (i) the opportunity for validation/replication of the main results and (ii) reuse of clinical trial data for secondary research. For the latter type the term “secondary research” specifies research based on datasets of already conducted clinical trials addressing research questions going beyond the original study protocol. Typically secondary research is conducted by independent research groups that were not involved in the original study team. The recommendation of analysis standards needs to be tailored to the respective objectives of the analyses.

### 2.1 The opportunity for validating the original study results and for investigating their robustness

The validation of the main results of a confirmatory clinical trial requires not only access to patient-level data, but also access to the clinical trial protocols (including amendments), statistical analysis plans together with software code, data dictionaries and the study reports. A reviewer pointed out that “software code may be proprietary information and hence challenging to share openly”. If the statistical analysis plan is detailed enough then the analysis may actually be reproduced even without knowledge of the original code. However, if this is not the case, access to the code might be essential to understand how the analysis was implemented. For validation purposes no prospective “validation protocol” seems to be necessary, as it will be guided by the original statistical analysis plan. However, the report on the validation should include all the necessary details to retrace the methodology applied, for example if additional sensitivity analyses are performed to check the robustness of the results of primary or secondary endpoints. Standards should not be prohibitive and go beyond requirements for the original data analysts. Still, in the first place this validation remains the responsibility of regulatory authorities in time during the assessment procedure. Some may even believe that this should only be done by regulators to avoid any confusion, others may not agree to such a monopoly of regulators. We appreciate that this initiative for transparency is backed by European regulators as re-analyses of the data may be the basis for criticism for regulatory decisions. Such discussions may increase the statistical workload and the current man power of statisticians within the European regulatory system is rather limited (Skovlund, 2009; Eichler et al., 2010). Some other regulatory agencies have historically approached this differently, for example, the US regulatory agency FDA has a large in-house statistics department that routinely re-analyses the data of pivotal trials.

### 2.2 Reuse of clinical trial data for secondary research

The scope of such research may range from “quasi prospective research” (with an analysis plan which was written without knowledge of the data) to full data mining, providing different levels of evidence. From a purely conventional confirmatory point of view research following publication of data and/or their analyses may be flawed by data-driven research targets or by an abundance of potential research targets, for example, with regard to different subpopulations and/or endpoints. Whereas in Phase III trials it is usually fairly easy to trace the time schedules of planning, performance and analysis, there is a completely different environment in secondary research.

- Higher levels of evidence will be provided by studies with a protocol written before access to the data. Meta-analysis based on individual patient data may be an example for such a higher level type of research. Another type of research is the analysis of stored specimen samples to investigate hypotheses arising outside the trials on the basis of the completed clinical data observed in those trials. However, generally when planning secondary research projects, there will be study results already available, either from publications, reports, or from other groups having access to the data, so that it will be difficult to exclude post hoc definitions of research objectives, for example, resulting in hunting for “statistical significance”. Early publication of protocols for secondary research, ideally before unblinded data of the original trials become available, would enhance credibility and persuasiveness of the planned secondary analyses. Ideally the same kind of rigor as used for the primary analysis should be applied, that is, a priori planned analyses, including well defined hypotheses and endpoints. In any case, the protocol and resulting publications should clearly refer to time lines of data access and background knowledge available when formulating the research objectives—even though this will be difficult to verify independently.
- Full explorative discovery using data mining methodology provides a lower level of evidence but may reveal new and useful results. These results in general will have to be confirmed by further research and it should be avoided that oversophistication of protocol standards in secondary explorative research disguise their limited level of evidence. Therefore, in all publications the nature of the research should be described clearly to allow an appropriate interpretation, for example, by prominently indicating the source of the data and its secondary use.

Overall, the quality of both validation and secondary research undertakings, will depend on the availability of datasets containing original measurements (in contrast to heavily preprocessed analysis datasets). Administrative hurdles to get access to the data should be minimized to avoid biased results due to incomplete access to datasets. For example, biased analyses may result if it is not possible to adjust for certain covariates as center or important variables to define analysis sets are not accessible. Furthermore, there is a trade-off between the range of statistical analyses that would be possible and the extent of anonymization of data. Anonymization inevitably reduces the information contained in the original dataset. For example, if variables such as dates, age (in days), or center, have to be removed for anonymization purposes, important covariates may be missing to perform validation as well as exploratory analyses.

## 3 Perspective from Jim Slattery

Many difficult questions are raised for regulators by requests for access to data arising in commercial clinical trials and residing within the agencies having been submitted by companies in support of a request for regulatory action. The rights of companies, clinical researchers, the patients from whom the data have been collected and the general public including, most importantly, the future recipients of the treatments studied in these trials must be balanced.

In the past, it should be admitted, the task of negotiating this minefield of potentially conflicting interests has resulted in some natural conservatism on the part of the European Medicines Agency in responding to such requests. The gradual moves away from this position at the EMA as regards to the release of information have culminated in a statement of intent by the director and chief medical officer of the EMA, together with some prominent members of the EU regulatory organizations (Eichler et al., 2012). The discipline of formulating such a statement is helpful for any public body because it forces it to concentrate on its specific responsibilities rather than try to satisfy all the opposing, and sometimes conflicting, interests. The agency has core values that are rooted in improvement of public health and it is a trivial observation that data that are hidden cannot be used by those from whom they are hidden to have any positive effect on health. Thus, promoting an open and transparent approach to dissemination of data would seem like a natural route for such an organization.

There is a strong ethical argument for wide access to clinical trial data. The patients who agree to participate in such trials do so in the hope that the information collected will improve the care of future patients. The responsibility of those holding the data must be to ensure every opportunity for this hope to be realized. Such data are very complex and the idea that one group of analysts, no matter how clever, will extract all the relevant clinical information is unrealistic. This is particularly true in the case of adverse events associated with the treatments, where the relevant questions to ask may not emerge until after the product has been on the market for some time.

The Agency, of course, recognizes that caution must be exercised. Patients in a clinical trial have both the right to believe that their data will be used to improve public health and also the right to expect that the data will not be used for other purposes and, most particularly, for purposes that are contrary to their interests. These latter purposes will usually involve identification of patients and hence measures are needed to ensure protection of sensitive personal data. It is also occasionally possible that release of some data from commercial trials might work to the disadvantage of companies. Both these possibilities should be minimized by responsible handling of release of data. However, in balancing the benefits and potential problems in release of data it is necessary to bear in mind that the most easily defensible interests may not be the most important from a societal viewpoint. The intention behind release of clinical data is to facilitate research that will help doctors to use medicines in a manner that reduces the risk of adverse reactions in patients and targets those likely to benefit most. However, as with many public health measures, those patients who benefit are not themselves aware of the processes that contribute to the success of their treatment. By contrast, anyone who may be adversely affected by release of data is identifiable and can appeal directly to many standard methods of redress. This imbalance in the visibility of benefits and risks is a familiar problem for agencies engaged in promoting public health and it is part of their job to point out the good that can be done by appropriate research and the necessity to use the often limited data to its maximum capacity.

A question also within the remit of the Agency is whether wide accessibility to clinical trial data could work against the interests of public health. The usual argument in this case is that multiple analyses could result in spurious findings that undermine confidence in good products and overburden those responsible for investigating them. This is particularly relevant with respect to drug safety because of the large number of possible adverse events that must be considered. Put simply, when we look at many different clinical outcomes some will, by chance, occur more often than others in any dataset. It is important to differentiate such random highs from true effects of the treatment. In the absence of any additional independent data, which could allow the question to be approached from a different angle, this decision is very difficult, often based on considerations of how the apparent effect was found, the support for a causal association in terms of the temporal relationship of drug exposure and outcome, and the current clinical knowledge of the mechanism of action of the product and similar products.

There are three reasons why the release of clinical trial data would not be expected to change markedly the current scenario with respect to drug safety. In considering these it is important to see such concerns within the context of the routine work of regulatory agencies. All the EU regulatory agencies monitor marketed products for emerging safety concerns using all sources of data available to them. This is already a very considerable body of data. At the EMA a large database of reports of potential adverse drug reactions, EudraVigilance, is the primary tool for finding new adverse drug reactions. In addition to the outputs of such databases as EudraVigilance, the regulatory agencies respond to safety signals emerging from independent research and from observations of clinical practitioners. The adverse events observed in randomized clinical trials are reported to another part of EudraVigilance, separate from the dataset for events observed in clinical practice settings, and hence some idea of the difference in scale of these data sources can be obtained. The number of reports received from clinical trials in 2012 was 34,174 while the number concerning the use of drugs following marketing was 536,228 (First Annual Report on EudraVigilance for the European Parliament, the Council and the Commission, 2013). On the basis of these figures the number of signals of safety problems arising from all clinical trials might be expected to be about 6% of the number arising from post marketing spontaneous reports and a still smaller percentage of the pharmacovigilance workload when other sources of safety signal are included. If we note that the clinical trials data released will not include all trials but only those communicated to the Agency as part of a regulatory submission, the potential impact begins to appear small indeed.

The second reason to believe the impact will be low is that most clinical trials have an in-built comparison group that can give a fair estimate of the numbers of adverse events for patients treated with alternative medicines, a feature that is absent in other data sources. Thus false signals do not only arise in other sources from chance imbalances (multiplicity) but also from systematic imbalances—for example, if patients treated with the new drug tend to have higher blood pressure than other patients they may have a high rate of coronary events and this may appear to be due to the treatment. Estimates of the current relative frequency of signals of real adverse drug reactions and false signals are available. A study using EudraVigilance (Alvarez et al., 2010) found that statistical methods routinely used to find drug safety problem unearthed at least seven potential drug safety problems for each adverse drug reaction discovered. Documentation of the proceedings of the group dealing with signals of drug safety at the Agency shows that the figure is even higher when we add the signals arising from other sources. Over the years 2009 to 2012 the pharmacovigilance group in the European Medicines Agency reviewed 7557 potential drug safety problems arising from a variety of sources. Of these 185 (about 1 in 40) were found to be genuine issues requiring further investigation in collaboration with Member State regulators and 48 (1 in 157) resulted in recommendations for a label change in the product concerned. Upon review many of these false-positives are found to result from systematic biases and so it is reasonable to expect that the rate of false-positive signals from clinical trial data would be far lower than this. Indeed the overall impact of making clinical trial data accessible should be to decrease the ratio of false-positive to true-positive signals.

The third reason for optimism is that the data in clinical trials will have been carefully monitored at every stage of collection and summarized in detail before submission to the regulators. Hence, many potential safety signals will already have been examined. Although EudraVigilance too is examined regularly the other major sources of safety data, clinical practice datasets, are only sporadically analyzed in response to hypotheses and hence are more likely to provide surprise results.

In summary, the addition of clinical trial data to the other available sources seems unlikely to greatly change the scale of the effort involved in monitoring drug safety but may improve the effectiveness of the activity. It will produce some additional false signals but the cost of finding true safety problems is the necessity of investigating such false signals. Few people would suggest that this cost is not worth paying.

In recent times the process started in 2010 has resulted in a draft policy on release of clinical trial data from the European Medicines Agency. This document attempts to address in a balanced fashion the many interests involved in access to such data. However, the over-riding theme is that the Agency believes that wider access to such data will promote public health.

## 4 Perspective from Trish Groves

### 4.1 Opportunities

There is much interest now in open science (The Royal Society, 2012). This is being driven by academic, scientific, regulatory, economic, and ethical imperatives as well as technological possibilities. Two of the biggest drivers are that the outputs of publicly funded research should be publicly available (http://pantonprinciples.org/) and that academic funding should not be spent on payments to get through journal paywalls. Moreover, open medical science has real potential to benefit patients and society, particularly if it allows further analysis of anonymized patient-level data from clinical trials which, in turn, increases the completeness, accuracy, and fidelity of the evidence base for medicines and other healthcare interventions (Altman and Cates, 2001; Eysenbach and Sa, 2001; Hutchon, 2001; Vickers, 2006).

For clinical trial participants data sharing will maximize their ability to make an altruistic contribution to science. But participants will need reassurances during the informed consent procedure that their privacy will be protected with a low risk of reidentification from the dataset—or, where that risk cannot be minimized, then their consent is sought on the basis that they are comfortable knowing that full anonymization will be impossible. For clinical trialists, knowing that the dataset will be available to others might ensure that data and analyses are gathered, curated, and archived with greater thoroughness; the temptation to report results selectively should be reduced; and scientific misconduct should be less likely. For methodologists access to such clinical trial data may allow testing of secondary hypotheses, development of novel statistical approaches, improvements in the design of future trials, and individual participant data (IPD) meta-analysis.

IPD meta-analysis has both statistical and clinical advantages and is growing in popularity (Riley et al., 2010). It is particularly valuable when meta-analysis of aggregate data cannot adequately answer the most important clinical questions, and when academics, clinicians, and policy makers need to detect differential treatment effects across individuals.

### 4.2 Practicalities and challenges

There are variable levels of openness in data sharing. Individual patient-level data may be available on request—either direct from the trial investigators and/or sponsors—or via a closed website from which data are released only after proposals for secondary analysis have been approved by a peer review process or panel (e.g., through the GlaxoSmithKline clinical study data initiative https://clinicalstudydata.gsk.com/). The panel discussion at MCP 2013 focused, however, on open access to clinical trial data. To be truly open, a dataset should be deposited and shared with a license that allows text mining and other forms of unrestricted reuse (http://www.plos.org/about/open-access/howopenisit/). And, as well as the anonymized patient-level data, the statistical code; details of the extent, nature, and handling of any missing data; and other supporting information should be available.

There are many concerns about confidentiality when sharing study data, but these should be surmountable with respect to both commercial confidence (EMA, 2013) and patient privacy (Hrynaszkiewicz et al., 2010; EMA, 2013). Another possible barrier is the pressure on academics to publish several studies using their data. This could be overcome by not sharing the dataset until after a pre-agreed period of fair use by the trialists. And whose data are they really, if a clinical trial was publicly and/or commercially funded and if the clinicians and patients who took part were in publicly funded healthcare settings?

Then there are the technical, ethical, and financial challenges of storing, curating, managing, and providing access to data in long term. However, repositories are burgeoning and academics increasingly have the support of data scientists in many scientific fields to ensure that they gather the data correctly in the first place and can then manage them properly.

### 4.4 Journal policies

Editors and peer reviewers may worry that they will be burdened by having to handle datasets as well as articles, but some repositories are making data sharing easy with deposition processes and timelines that are integrated with article submission and with linkage back to the related journal articles (e.g., article submission to *BMJ* Open is integrated with data deposition at Dryad http://datadryad.org/discover?field=prism.publicationName_filter&fq=location:l2&fq=prism.publicationName_filter%3Abmj%5C+open%5C%7C%5C%7C%5C%7CBMJ%5C+xOpen). Meanwhile a new generation of journals, dedicated to publishing datasets, is growing fast. NPG's Scientific Data is one such journal (http://www.nature.com/scientificdata/about/).

Some medical journals’ policies are actively encouraging authors to share data. Since 2009 Annals of Internal Medicine has asked authors to include in their manuscripts reproducible research statements (Laine et al., 2007), a policy in turn inspired by that of Biostatistics (Peng et al., 2006). The *BMJ* also introduced data sharing statements for all research papers in 2009 and, in January 2013, went further by bringing in a policy of considering trials of drugs or medical devices only if the authors agree to make the anonymized patient-level available on reasonable request (Godlee and Groves, 2012).

The *BMJ* is also a cofounder of the AllTrials initiative in January 2013, whose online petition for all trials to be registered and all results reported attracted in its first 6 months more than 50,000 signatures and endorsement by more than 80 patients’ groups and many major academic and professional bodies (http://www.alltrials.net/). Although the panel discussion at MCP 2013 was largely about patient-level data, it was worth pointing out in the session that incomplete compliance with what should be much lower hurdles—trial registration and reporting of all summary results—are still blocking access to much trial information.

Researchers aiming to ensure the integrity of the therapeutics evidence base are increasingly turning to previously confidential company research documents such as Clinical Study Reports. These have been released through freedom of information requests to medicines regulators, or through legal action. What should such researchers do if they find in these documents details of clinical trials that were started but either unregistered, unfinished, unpublished, or a combination of all three?

One group of researchers is calling for sponsors and investigators to publish such invisible or abandoned studies, and is proposing a system for independent publishing if these calls go unanswered (Doshi et al., 2013). This system for restoring invisible and abandoned trials (RIAT) comprises: (i) obtaining clinical study reports and any other study data, (ii) collecting documentation of trial abandonment, (iii) issuing a “call to action” by publicly registering their possession of data sufficient for publication, (iv) collecting documentation of the need for restoration of the trial to the literature, (v) presubmission inquiry to a “RIAT friendly” journal willing to consider articles, (vi) preparation and submission of manuscript according to RIAT procedures.

As the RIAT authors acknowledge “Some people may think that publications based on clinical study reports with which the authors have no connection is equivalent to intellectual property theft, but you cannot steal what is already in the public domain (and only in the public domain because a drug regulator or judge had the documents unconditionally released or the sponsor waived their confidentiality claims over the documents). The considerable discussion about the need for public access to trial data and data ownership has not yet resolved how to handle the thorny but important question of proper scientific credit … RIAT authors would be able to claim credit for bringing to light what was previously invisible or distorted but not for carrying out the trial”.

The editors of both the *BMJ* and *PLOS Medicine* have endorsed the *RIAT* proposal, committed to publishing restorative clinical trial submissions, and called on other journals to do the same (Loder et al., 2013).

## 5 Perspective from Thomas Lang

In their draft policy/0070 (EMA, 2013), EMA states that “transparency is a key consideration for the Agency in delivering its service to patients and society”. Although transparency is beyond any doubt a worthwhile goal to be followed, it appears crucial to highlight some of the probable implications of a transparency initiative in relation to publication of clinical trial raw data. Especially some untoward implications might not be obvious at first sight, but could be both, major and undesired, if not considered and adequately addressed in advance.

Section 2013 introduces two types of statistical analyses which would be triggered by the policy. The first (i) being independent replication of clinical trial data analysis to verify the regulatory authority's positions and challenge them where appropriate, and the second (ii) related to secondary analyses enabling the wider scientific community to make use of detailed and high-quality clinical trial data to develop new knowledge in the interest of public health.

The first goal (i) needs to be seen in context of the current practices of assessment work and regulatory decision making in the framework of centralized marketing applications. At the level of EMA/CHMP, decision making in relation to licensure of new drugs (positive/negative opinion) is currently based on rapporteurs’ assessment work without access to clinical trials data on patient level in electronic format. Assessment is based on thorough review of protocols, analysis plans and clinical trial reports, and usually does not involve processing of patient raw data to replicate analyses carried out by the Sponsor/Applicant. This assessment approach is considerably different to that of the US-FDA, where electronically processible trial raw data are usually part of the application dossier, and where substantial resources are allocated to data analyses replication on the regulatory side. The current European approach, however, is to assess traceability, that is, congruence with the predefined analysis plan and internal consistency of study results, the suitability of the methodology and the interpretation of the study results. If gaps or doubts are identified during assessment, additional analyses are requested from the Sponsor to clarify potential issues.

The on-going discussion on Clinical Trial Data Access has a flavor of distrust in this current European regulatory review and decision making process. Any concerns and doubts in this regard should be taken seriously, but also the question should be raised if a policy on raw data access is the right answer to address such concerns. If there is actually that much of distrust in the current system, it would seem worthwhile to think about a change of the assessment/decision making paradigm anyhow, irrespective of an open data policy. As mentioned in Section 2013, the current man power of statisticians within the EU regulatory system is rather limited. About 25 qualified statisticians are available in the whole regulatory system for assessment work, most of them employed by national agencies. This limitation is also reflected by the fact that unfortunately not all assessment teams nominated by rapporteurs for centralized procedures include a statistician/methodologist. In light of this, the logical first step is not to start an initiative that calls for (external) re-analysis of study results, but would be to ensure sufficient allocation of methodological expertise in the assessment of marketing authorization applications.

However, in the GCP environment as established today, one might not consider the re-analysis of study results as the primary issue within the framework of the assessment of a marketing authorization application. Instead, of key relevance for the regulatory system is an assessment of the study design, the appropriateness of the analysis strategy and the interpretation of study results. The utilization of raw data can by no means replace any of the methodological assessment done so far in the European regulatory system.

Another important question in this context is whether sole replication of clinical trials’ data analyses makes it really possible to (fully) understand the regulator's decision. The decision making process in relation to licensure of a new drug is becoming more and more complex, and decisions are based on risk-benefit evaluations across all sources of evidence brought up during the whole drug development program. Usually, a larger set of preclinical and clinical trials informs the regulators’ decision, and it may be worthwhile to think about other measures (than granting access to raw data) to improve transparency in relation to decision making.

In case clinical raw data from registration trials for licensed products would be (re-)analyzed in the public domain, it is likely that (potentially conflicting) results from such analyses will be published and communicated via many different communication channels, most of the time outside control of regulatory bodies. As there are currently no systems in place to provide any kind of “arbitration” between potentially deviating/conflicting results, it appears to be of paramount importance (and needs to be understood as current responsibility of EMA and national agencies) that, in such a scenario, the question of responsibility for adequate patient information and corresponding risk communication would be adequately addressed within the framework of a transparency policy. This actually appears to be the most challenging task of all, not least because of the (anyhow) limited resources for methodological assessment on the regulatory side (as mentioned above).

With regard to the second goal (ii) mentioned above, a potential benefit for patients and prescribers from making clinical trial data accessible will only be achievable if third parties with sufficient technical and methodological capacity would do such secondary data analyses. The EMA policy would in principle enable requesters to merge datasets from different trials and to generate pooled datasets. This could allow for new meta-analytic approaches to further explore specific hypotheses and questions, which were left un-answered within the individual drug licensing dossiers (e.g., safety issues). Making use of clinical trial data to generate new hypotheses and/or to further explore open issues in relation to new (un-)marketed drugs can be seen as one of the biggest opportunities after implementation of the EMA policy. The draft policy encourages requesters to plan such analysis as early as possible, ideally before requested data material becomes accessible for the requester to ensure credibility of subsequent results.

The range of secondary analyses by making use of clinical trial data may, however, also include analyses based on data coming exclusively from one particular trial, with the goal to add new or challenge existing claims. In this context, the question is what kind of analyses would be informative and beneficial if one—at the same time—wishes to respect the methodological principles usually applied in drug development and regulatory decision making, that is, adequate control of the probability of a false-positive decision on the basis of one particular set of experimental data coming from one specific clinical trial? It is considered good clinical practice that the strategy and methodology for data analysis of a particular trial are laid down in the study protocol and analysis details are further described in a corresponding statistical analysis plan, which is prepared in advance of trial commencement (or prior to data base lock/unblinding). It has to be noted that the view on the importance of predefinition of statistical analysis strategies is commonly shared by regulators and sponsors and also by the scientific community, and is laid down in respective ICH guidance. This is not only to avoid arbitrariness in method selection at any later stage, but also (and in particular) to safeguard decision makers against an inflated risk for false-positive decisions, caused by any kind of unplanned or even data-driven post hoc data analysis. There is common agreement that as soon as an analysis plan is specified, it is—from the methodological perspective—important not to deviate from this plan and not to pursue a different strategy post hoc. This would apply to any party, be it the sponsor, a competitor, academia, or a regulatory agency. Third party experts could think of fully suitable alternative statistical analyses, but could also bring in a wide range of inferior approaches. Each scenario can result in a series of analyses leading to different results. The field is wide open to search the data for meaningful (and meaningless) subgroup analyses or to apply favorable (or unfavorable) approaches to impute missing data. Multiplicity opens enormously wide dimensions in this context, with an almost infinite number of thinkable ideas, and also with clear potential for conflicting results coming from analyses conducted by different requesters. Making any decisions on such post hoc data analyses clearly contradicts the trial-inherent concept of (strong) control of the family-wise type I error. Additional post hoc decisions may also bear the risk for false-negative conclusions. Against this background, having statistical analysis plans of such planned individual secondary analyses centrally available can naturally not cope with the requirement of full type I error control. Adequate control of type I error probability over all potential secondary analyses on one and the same dataset—if possible at all—would call for a sophisticated overarching statistical concept (as one option, see comment/proposal concerning FDR by Yoav Benjamini). The current draft version of the EMA policy leaves this aspect entirely open.

During the panel discussion, another open issue became evident, namely the potential conflicts between different raw data transparency initiatives worldwide. Whereas in some regions (e.g., the EU) a quite liberal data access system might be established, regulatory bodies of other regions may opt for more restricted access systems (e.g., FDA proposal to make masked data available presented by Sue-Jane Wang at the MCP's panel session). Conflicts could especially appear in relation to data transparency for multiregional trials, for example, trials with recruiting centres in the EU and the United States, where these trials would be relevant for licensing in both regions. Also, similar initiatives by other organizations, such as scientific journals (e.g., *British Medical Journal*, see comment by Trish Groves) or pharmaceutical companies (e.g., GlaxoSmithKline, see Nisen and Rockhold, 2013) would need to be considered. Given this background, it may well be that (e.g.) secondary publications after data sharing may become available in one region of the world before the regulatory review process in another region is finished at local health authority level. Implications of such situations on regulatory assessment in an ongoing marketing authorization procedure are currently difficult to predict.

## 6 Perspective from Yoav Benjamini

Three important issues are always addressed by the primary statistical analysis of confirmatory clinical trials.

- Forms-to-Figures reproducibility.
- A priori defined statistical analysis, meaning that the choice of endpoints for success and safety, the statistical procedures, treatment of missing values, and all other computational details will be fully specified before the analysis is conducted.
- Appropriate statistical analysis. This requirement has many facets but an important one is the use of methods that address the effect of multiplicity over the predefined family of inferences.

The first requirement is well answered by the EMA draft policy (EMA, 2013). The issue of apriori defined statistical analysis is echoed by proposing: “*the Agency considers preparation and uploading of a detailed protocol/statistical analysis plan before data access of utmost importance, to ensure the credibility of subsequent results*”. However, one of the benefits of secondary analyses of an open database is the offered flexibility in the process. Research questions will hopefully follow one from the other, and it will be very difficult, and possibly ineffective, to specify all details ahead of analysis. Moreover, different researchers may ask the data similar questions, or entirely different questions. They may all ask these questions on the same data or data that grows in size and complexity. Hence, the family of inferences will not be well defined. The appropriateness of the statistical analysis has not yet been addressed at all, until the recommendations will be detailed.

Unlike other statistical issues, where solutions are mostly available and agreed upon (at least at the basic levels) a methodology that is appropriate for addressing the effect of multiplicity in such free form investigations are still in the making, and the knowledge about the availability of some is not widespread. To state just a few concrete questions: How do we address multiplicity in a single study, where the inferential frame is not predetermined, or at best not well determined? How do we assess the effect of selection across different studies? How should long follow-up analyses be done? I will therefore devote most of my comment to present venues of analysis that allow free exploration of the data, while using appropriate statistical methodologies that will “ensure the credibility of subsequent results”; as required.

Controlling the Family-Wise Error Rate (FWER) is quite an impossible task, because the families of inferences are not predefined. Sometimes one can define a mega-family, the selections from which will include all other inferences that may become of interest. This approach can become prohibitively powerless. An example is the post selection inference presented by Prof. Buja at the MCP 2013 conference, where the inference is valid for any selected regression model because it controls the FWER for all possible ones.

The scalability of FDR control, and its dual False Coverage-statement Rate (FCR) offer a reasonable way to address the result of selective inference. They can be easily implemented in a single study with predefined family of interest. I will now discuss a few approaches that allow us to control the FDR with multiple families and/or multiple studies, as well as studies with increasing set of hypothesis as those that may be mined from an open database.

### 6.1 Sequential studies of a single database

Foster and Stine (2008) suggest the following setup: Consider a sequence of studies, where in study *i* hypothesis *H_i_* is tested. It is desired to control the mean FDR. Namely, if *R* is the number rejected, and *V* of them are rejected in error,


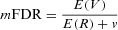


for some small ν, over the entire sequence of tests, which need not be determined beforehand. They also suggest to test *H_i_* by comparing its *p*-value with α*_i_*; but if there was a rejection in the *i*-th study α*_i_*_+1_ > α*_I_*, offering greater power if there was a rejection (because the denominator increases). They gave a simple and effective rule for choosing and modifying the constants α*_i_*. Aharoni and Rosset (2013) generalized the rules, and even found an optimal one. They also combine it with a database quality preserving scheme, where each user of the database pays (by adding data or its equivalent in price to the database) so that the quality in terms of power is maintained.

Note, however, that the order still needs to be maintained by the keeper (administrator) of the database, who assigns the α*_i_* to the *i*-th investigator.

### 6.2 Hierarchical studies: FDR control on the average over the selected families

**Figure 1:**
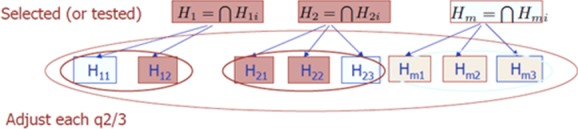
Testing a family of families (Benjamini and Bogomolov, 2014). Based on the data at hand H_m_ is not selected, so H_m1_, H_m2_, and H_m3_ are not tested. Since two of three families were selected at each family FDR needs to be controlled at level q(2/3) in order to assure FDR on the average over the selected at level q.

In a complex study one can consider many families of hypotheses. These can be a family of primary safety endpoints, of secondary safety endpoints, of efficacy on subgroups, and so on. In hierarchical analysis one infers on a family only if it is selected, say by testing the intersection of the hypotheses in each family (but not necessarily so). Figure 1 shows a schematic presentation of this structure. The testing scheme of Benjamini and Bogomolov (2014) described in the Fig. 1 is proven to control the FDR on the average over the selected at level q, as long as the selection is “simple” in a well-defined way. Moreover, if we consider any single secondary study of efficacy as a family this method can be used to assess its findings in light of the other ones that have taken place.

### 6.3 Hierarchical studies: Overall FDR control

With an open database, analyses are expected to accumulate and expand with the progression of time. A discovery in one study generates new research, with its multiple hypotheses, while a nondiscovery is not followed. Yekutieli et al. (2006) offer this testing scheme, as schematically displayed in Fig. 1, for the analysis of a single even if complex study. They suggest to test each subfamily of a parent discovery by the Benjamini-Hochberg procedure at FDR level q.

Yekutieli (2008) proved the following surprising property: for any size and shape of the tree, if the parents are independent of the hypotheses just below them, then independent FDR <2cq, with c < 1.44 but usually close to 1. It means that secondary analyses can accumulate in an unplanned way—and multiplicity can still be addressed if each study controls the FDR and the parent of many similar studies is significant in meta-analysis.

### 6.4 Summary

When addressing in the same study many research questions (even if added during the analysis), control for multiplicity (at least) using FDR should be required. This should be written into the “good analysis” recommendations!

When independent studies have accumulated, all testing the same scientific hypothesis, or its close variants, each controlling the FDR, meta-analysis is in need, where all results pertaining to similar hypothesis are collected, and assessed jointly while controlling for multiplicity. This also allows the assessing of the replicability of the results.

**Figure 2:**
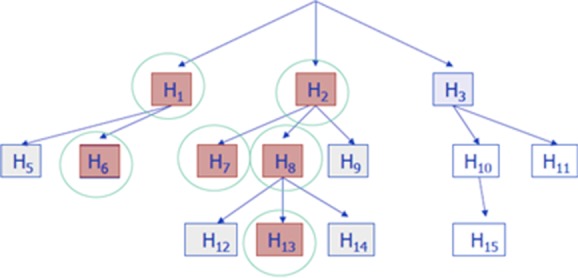
Hierarchical testing of hypotheses (Yekutieli et al., 2006). Each family of hypotheses is tested at FDR level q only if its parent hypothesis was rejected. Counting all discoveries made, the overall FDR remains at about 2q, no matter how large and what form the tree takes.

An alternative approach is to require each new study to collect all similar studies and do a “local” meta-analysis, to ensure that its “identified parent” is tested and rejected at level q. If this is judged to be too demanding, inspecting the hierarchical structure along the lines in Fig. 2 will allow the Agency to assess jointly the importance of the secondary analyses’ results.

## 7 Perspective from Simon Day

We might ask: “Why don't we routinely allow open access to data?” Do we then enact some self-criticism for not having been arguing this position for many years, and should we simply be accused of jumping on what has become the politically correct band wagon? Perhaps both of these, but it seems quite clear that times change and what may (and we emphasize “may”) have been acceptable in years gone by seems unacceptable today. We argue this from two perspectives: first that it seems self-evidently quite reasonable for patients, prescribers, payers, and all others, to know as full and complete information about medicines that are in everyday use as can be known; and second that, sadly, it is clear that not everyone tells the truth, the whole truth, and the whole truth the whole of the time.

The extent of outright fraud is extremely difficult to gauge, but it seems not immaterial (Buyse et al., 1999). There are clear, documented cases of fraud in academia as well as in the private sector (Lock and Wells, 1993). Probably unfairly, intentional deceit from within the pharmaceutical industry seems to attract much more high-profile public criticism than similar intentional deceit emanating from academia. But aside from intentional deceit, there is a probably much more prolific problem of lack of competence—both in terms of technical skills to analyze and report data, as well as the more philosophical challenges of understanding statistical concepts. Multiplicity is surely one such area full of philosophical challenges. Many researchers can barely understand what a *p*-value less than around 5% might mean, but how can the meaning of that *p*-value be affected by some other analysis done on the same dataset? Aren't the data just that: that data? Consider the uncomfortable position of having to explain to a colleague why his small *p*-value calculated at the end of a clinical trial that had included an interim analysis with a group sequential stopping rule (that had not been met) could not be considered as “statistically significant” because of the interim analysis that was carried out a year ago on the first half of the data. In response to the retort: “But I never wanted to include the interim analysis anyway”, it is difficult not to accept that the colleague's trial has to be interpreted based on the whim of a design aspect someone else incorporated. Statistics (certainly from a frequentist point of view) seems to have set itself up to include a lot of challenges.

So, it is probably irrelevant whether simply “changing times” is an adequate reason to be making such fundamental changes to how we have worked for many years, or whether an accumulation of misinformation (intentional or otherwise) has led us to recognize that something has to change. Times *are* changing; misinformation *is* happening. We need to accept these facts but then consider where we need to be cautious (e.g., multiplicity), and what safeguards need to be in place. The safeguard of preventing erroneous data analysis (including problems of multiplicity) by keeping the data hidden is not tenable.

Licensing of new medicines hinges on understanding and balancing benefits and risks. Certainly not all clinical trials (let alone clinical research) are done with a view to licensing—nor even evaluating—new medicines, medical devices, medical procedures, and so on. But let us consider the balance of benefits and risk. Every clinical trial intended to compare the effects of two or more interventions runs the risk of getting the wrong answer, simply through the play of chance. Every analysis of a patient registry, or survey analysis, runs a similar risk. These risks are inherent whether we adopt a hypothesis-testing approach or some other philosophy. Whatever we conclude from our data might be wrong, but this does not prevent us from striding ahead and carrying useful and important clinical research. We need to take a similar approach to open access to data and the possibilities of multiple follow-on analyses and their potential for false-positive—and false-negative—findings. How valuable would it be to know what has already been done, what the results were, and what questions are left not fully resolved? What level of disaster would strike us if some further analyses from those datasets came to wrong conclusions? Much of the answer to the question of “how much risk” rests in who is looking at the data. A cynic might suggest that the reporters for our daily newspapers are not capable of understanding the subtleties of multiplicity. So why not give them a chance? Like most of us, many of them might learn from their early mistakes but with the seemingly near infinite array of new analyses that are envisaged to flow from these new found (or newly released) data, we will soon tire of reacting too quickly to every alarm signal. After an initial hiatus (which will probably happen), we will soon come to balance the benefits of various features of datasets, including size of data, source of data, quality of data, strength of effect, plausibility of effect, reproducibility of effect, as well as issues about prespecification and multiplicity adjustments.

Amongst this early hiatus, there will probably be a flood of bad analyses, aimed solely at “proving” the analysts’ pet theories. These will include belittling the findings of others, including the findings of results of clinical trials carried out by the pharmaceutical industry. This will not be good for the pharmaceutical industry but—much more importantly—it will not be good for the public health. Patients will be left confused and worried. Nor will it be good for public health for a similar raft of activity to prove an analyst's pet theory about the benefits of some potion in some vague indication. We have already seen consequences for public health of such chasing after pet theories with the report by Andrew Wakefield and colleagues of a link between autism and MMR vaccination. Scientifically this was quickly discredited but it took many years for vaccination rates to return to their pre-Wakefield levels and many babies were harmed during that time. Many families have suffered, and continue to do so as a result. Such risks need to be balanced against the potential gains coming from broad teams of scientists being able to investigate datasets for new findings. Such risks also need to be balanced against the potential gains coming from broad teams of scientists being able to disclose “covered up” (or, generously speaking, “missed”) findings in clinical research data: for example, the types of disclosures identified—but with much more difficulty than if the raw data had been available—by Jureidini et al. (2008).

The risk, and particularly the consequence, of false safety signals is important (as Slattery has pointed out earlier in this article). What about the risks of false findings of apparent efficacy? Here the consequences, in many cases, may be less extreme. A therapy that apparently seems to work in some indication (or in some subpopulation) where it was previously either believed not to work, or had never been looked at, may simply be the result of a Type I error: a clear multiplicity issue. But of the Type I errors falsely apparently showing that a treatment works, in how many cases might that treatment actually be harmful (as Schwartz et al., 1980, would call it, a “Type III error”)? Likely it will be very few and patients will simply be taking placebos: not something to be advocated, but not as serious as many other Type I errors. The areas where Type III error does raise greater concern is in situations of clear and important unmet medical need. In life-threatening conditions, where no proven therapy is available, a Type I error (but unknown to all of us that it is such an error) will give patients false hope and likely impede the progress of developing new therapies. Patients and their physicians may be reluctant to take part in good quality prospective clinical trials when an apparent therapy already exists. Why would I opt to be randomized to an experimental therapy which might work, when there is a “known” therapy already available to which I have access?

## 8 Concluding remarks

Even though statistical decision criteria may lose their meaning in secondary research—which might be a painful experience for our profession—there is no way back to the days where only the sponsor, but no one else, had access to the raw data. If following the scientific principles implies not to perform any secondary research at all, this should be a reason to question the scientific principles in the first place. In very early times of clinical trials some people argued that in the situation of equipoise the informed consent of the patient may weaken the scientific rigor of the trial because of potentially biased samples, early dropouts, patients' knowledge about possible side effects, and so on. Nevertheless, today the importance of the autonomy of patients' decisions is not questioned—and for good reasons. Although secondary research may deviate from “pure science”, this should not be seen as argument to prevent transparency in this sensitive area where so many different interests are focusing.

Transparency is considered to be a positive feature in areas of public interest—or in areas that then become of public interest. However, the limits of transparency depend on the political and legal environment in a society. For example, only a small proportion of all the data that are behind the publications in *BMJ* are, in fact, covered by its open access to patient-level clinical trial data initiative. Furthermore, it would be interesting to get more details on the legal arguments behind the very cautious approach to data sharing of the US FDA and to know whether the well written draft of EMA policy/0070 is aiming at maximal transparency simply because it is expected to be cut down later anyway.

In principle incomplete reporting of data to health authorities cannot be repaired directly by making the incomplete information public. But whistle blowing by persons having been involved in the generation, handling or analysis of potentially missing information may help to identify such cases. Clearly this may be an incentive to ensure accuracy of datasets and to reduce deliberate misconduct.

The regulatory decision process can be checked in one way or other, which may reach from simply repeating analyses to adding new analyses, for example, with regard to homogeneity over subgroups. Obviously such checks should be done before important decisions are taken and therefore should be within the remit of the health authorities. Thomas Lang has asked whether in Europe there is distrust in current decision making and whether a change in the assessment paradigm is required. From fairly recent surveys (Skovlund, 2009) it is obvious that the manpower and know how in data handling, modeling and statistics at the European regulatory agencies is very limited. However, the few statistician working at the EMA or national agencies may expect a lot of work: When the open data initiative comes into force, immediately rapporteurs and reviewers will have to consult statisticians on a regular basis so as not to be on their own when assessing design and analysis issues. Maybe in future more statisticians and data managers will be hired by European regulatory agencies. The leading regulatory persons behind the initiative seem to aim at improvement of regulatory decisions within the dispersed bureaucratic European system: We should not forget that access to clinical trial data in their wording should “enable third parties to verify the regulatory authority's positions and to challenge them where appropriate”.

Reuse of data or secondary research is an important methodological aspect of the change in policy. However, there is a major caveat: the innocence of the planned study paradigm laying down the main inference features in advance will be lost in secondary research. Who—in this area where so many conflicting interests are focusing—will be able to exclude data-driven analyses, as soon as the data have been made public? What, in secondary research, is the meaning of error rates and what is the role of a study protocol, how can we assure that the level of “preplanning” can be properly assessed and honestly transmitted in publications? Here, a huge area of “multiplicity” may arise from high-quality datasets and we may not even realize they exist when hunting for saleable results strategies (saleable in scientific or economic terms). At this point, again, scientific journals may have to take some responsibility. Koenig, Bauer, and Posch explored the opportunities for validation and secondary research that arise with access and sharing clinical trial data on patient level. Slattery noted that multiplicity is a fact of life in drug safety so that there we can live with a bit more. Lang is concerned about the level of inference, and we may question the extent of public health risks of false conclusions following from secondary research—clearly without being able to give final answers. Benjamini gave us the hope that the concept of the FDR may help to achieve some level of inference and further details on how to apply this here would be very interesting. Also the need for confirmation and the important role of meta-analyses has been mentioned, for example, Wang addressed statistical problems in cumulative meta-analyses during her presentation at the MCP meeting. And why should we not expect interesting results and hypotheses form secondary research in large (clinical) data bases collected in a costly and time consuming way? Is it not a great opportunity, particularly for the academic area? If our philosophy of science would deny such a secondary research in principle, we should reconsider our philosophy of science.

Data sharing may be of particular importance for small populations where generating clinical evidence is very challenging, for example, in the development of orphan drugs, personalized medicines, or drug development for children. Patient-level data of already conducted trials may enable the identification of patient subgroups that benefit most, may serve as historical controls, may facilitate meta-analyses of small trials, and may be used to formulate prior distributions in Bayesian analyses. By having access to historical clinical trial data, one can develop tailored statistical models at the planning phase that make best use of the newly generated data. For example, one can optimize the measurement time points or select relevant covariates in advance. However, though small population research may benefit greatly from open access to clinical trial data, it is also the area where the risks with regard to patient privacy are highest.

We are convinced that the scientific community will learn how to properly deal with the planning, data protection, analysis, publication, and most importantly the interpretation of secondary research, and so will the public. However, we acknowledge that the communication of results coming from secondary use to the public domain may become the most delicate problem in the whole environment. For secondary research there is also a mandate to publish also negative results to avoid publication bias.

But for the moment let us wait for what the lawyers will say, because there seems to be a general tendency that political decisions on the appropriateness of innovative moves in societies are handed over to lawyers and courts.

## Authorships and disclaimer

The authorships of Sections 2 to 7 are indicated in the section titles. For Section 8 the authorship is shared between Peter Bauer, Yoav Benjamini, Simon Day, Trish Groves, Franz König, and Martin Posch. Jim Slattery: The views expressed in this article are the personal views of the author and may not be understood or quoted as being made on behalf of or reflecting the position of the EMA or one of its committees or working parties. Thomas Lang: The views expressed in his chapter are personal views of the author and may not be understood or quoted as being made on behalf of or reflecting the position of the Austrian Competent Authority. Previous versions of Sections 2 and 8 have been submitted to the EMA advisory group on good analysis practice for their notice. Franz Koenig's and Peter Bauer's research has received funding from the European Union Seventh Framework Programme [FP7 2007-2013] IDEAL under grant agreement No.: 602552. The research of Yoav Benjamini was supported by an ERC Advanced Researcher Grant (PSARPS). Simon Day's research has received funding from the European Union Seventh Framework Programme [FP7 2007-2013] InSPiRe under grant agreement No.: 602144. Martin Posch was supported by the EU FP7 HEALTH.2013.4.2-3 project Advances in Small Trials dEsign for Regulatory Innovation and eXcellence (Asterix): Grant 603160.

## Footnotes

1 EMA website on release of data from clinical trials see http://www.ema.europa.eu/ema/index.jsp?curl=pages/special_topics/general/general_content_000555.jsp&mid=WC0b01ac0580607bfa

## References

[b1] Aharoni E, Rosset S (2013). Generalized Alpha Investing: Definitions, Optimality Results, and Application to Public Databases. Journal of the Royal Statistical Society Series B.

[b2] Altman DG, Cates C (2001). Authors should make their data available. British Medical Journal.

[b3] Alvarez Y, Hidalgo A, Maignen F, Slattery J (2010). Validation of Statistical Signal Detection Procedures in EudraVigilance Post Authorisation Data: a retrospective evaluation of the potential for earlier signalling. Drug Safety.

[b4] Benjamini Y, Bogomolov M (2014). Selective inference on multiple families of hypotheses. Journal of the Royal Statistical Society Series B.

[b5] Buyse M, George SL, Evans S, Geller NL, Ranstam J, Scherrer B, Lesaffre E, Murray G, Edler L, Hutton J, Colton T, Lachenbruch P, Verma BL (1999). The role of biostatistics in the prevention, detection and treatment of fraud in clinical trials. Statistics in Medicine.

[b6] Doshi P, Dickersin K, Healy D, Vedula SW, Jefferson T (2013). Restoring invisible and abandoned trials: a call for people to publish the findings. British Medical Journal.

[b7] Eichler H-G, Abadie E, Breckenridge A, Leufkens H, Rasi G (2012). Open clinical trial data for all? A view from regulators. PLoS Med.

[b8] Eichler HG, Hemmings RJ, Vamvakas S (2010). Where Statisticians can Contribute to Address Future Challenges for Drug Regulatory Agencies: A View from the European Medicines Agency (EMEA). Statistics in Biopharmaceutical Research.

[b9] EFPIA and PhRMA (2013). http://transparency.efpia.eu/uploads/Modules/Documents/data-sharing-prin-final.pdf.

[b10] EMA (2010). http://www.ema.europa.eu/docs/en_GB/document_library/Other/2010/11/WC500099473.pdf.

[b11] EMA (2013). http://www.ema.europa.eu/docs/en_GB/document_library/Other/2013/06/WC500144730.pdf.

[b12] EMA Press Office (2013). http://www.ema.europa.eu/docs/en_GB/document_library/Press_release/2013/12/WC500158390.pdf.

[b13] Eysenbach G, Sa ER (2001). Code of conduct is needed for publishing raw data. British Medical Journal.

[b14] FDA (2013). http://www.gpo.gov/fdsys/pkg/FR-2013-06-04/pdf/2013-13082.pdf.

[b15] First Annual Report on EudraVigilance for the European Parliament, the Council and the Commission (2013). http://www.ema.europa.eu/docs/en_GB/document_library/Report/2013/07/WC500146607.pdf.

[b16] Foster DP, Stine RA (2008). Alpha-investing: a procedure for sequential control of expected false discoveries. Journal of the Royal Statistical Society Series B.

[b17] Godlee F, Groves T (2012). The new BMJ policy on sharing data from drug and device trials. British Medical Journal.

[b18] Hutchon DJR (2001). Infopoints: Publishing raw data and real time statistical analysis on e-journals. British Medical Journal.

[b19] Hrynaszkiewicz I, Norton ML, Vickers AJ, Altman DG (2010). Preparing raw clinical data for publication: guidance for journal editors, authors, and peer reviewers. British Medical Journal.

[b20] Jones CW, Handler L, Crowell KE, Keil LG, Weaver MA, Platts-Mills TF (2013). Non-publication of large randomized clinical trials: cross sectional analysis. British Medical Journal.

[b21] Jureidini JN, McHenry LB, Mansfield PR (2008). Clinical trials and drug promotion: Selective reporting of study 329. International Journal of Risk and Safety in Medicine.

[b22] Laine C, Goodman SN, Griswold ME, Sox HC (2007). Reproducible research: moving toward research the public can really trust. Annals of Internal Medicine.

[b23] Lock S, Wells F (1993). Fraud and Misconduct in Medical Research.

[b24] Loder E, Godlee F, Barbour V, Winker M (2013). Restoring the integrity of the clinical trial evidence base. British Medical Journal.

[b25] Nisen P, Rockhold F (2013). Access to patient-level data from GlaxoSmithKline clinical trials. New England Journal of Medicine.

[b26] Peng RD, Domenici F, Zeger SL (2006). Reproducible epidemiologic research. American Journal of Epidemiology.

[b27] Riley RD, Lambert PC, Abo-Zaid G (2010). Meta-analysis of individual participant data: rationale, conduct, and reporting. British Medical Journal.

[b28] Schwartz D (1980). Clinical Trials.

[b29] Skovlund E (2009). Statisticians in European regulatory agencies. Pharmaceutical Statistics.

[b30] The Royal Society (2012). http://royalsociety.org/uploadedFiles/Royal_Society_Content/policy/projects/sape/2012-06-20-SAOE.pdf.

[b31] Vickers A (2006). Whose data set is it anyway? Sharing raw data from randomized trials. Trials.

[b32] Wei L, Bretz F, Hsu J, Posch M (2014). Editorial MCP 2013. Biometrical Journal.

[b33] Yekutieli D (2008). Hierarchical false discovery rate controlling methodology. Journal of the American Statistical Association.

[b34] Yekutieli D, Reiner-Benaim A, Benjamini Y, Elmer GI, Kafkafi N, Letwin NE, Lee NH (2006). Approaches to multiplicity issues in complex research in microarray analysis. Statistica Neerlandica.

